# Effect of Yangtze River on population genetic structure of the relict plant *Parrotia subaequalis* in eastern China

**DOI:** 10.1002/ece3.1734

**Published:** 2015-10-05

**Authors:** Qifang Geng, Zhigang Yao, Jie Yang, Jia He, Danbi Wang, Zhongsheng Wang, Hong Liu

**Affiliations:** ^1^ College of Life SciencesNanjing University22 Hankou RoadNanjing210093China; ^2^ State Key Laboratory of Pharmaceutical BiotechnologyNanjing University22 Hankou RoadNanjing210093China; ^3^ Wetland Conservation Station of Jiangsu Province22 Dinghuaimen StreetNanjing210036China; ^4^ Department of Earth and EnvironmentInternational Center for Tropical BotanyFlorida International UniversityMiamiFL33199USA; ^5^ College of ForestryGuangxi UniversityNanning530004China

**Keywords:** Genetic structure, ISSR, *Parrotia subaequalis*, relict plant, Yangtze River

## Abstract

*Parrotia subaequalis* (Hamamelidaceae) is a Tertiary relic species endemic in eastern China. We used inter‐simple sequence repeat (ISSR) markers to access genetic diversity and population genetic structure in natural five populations of *P. subaequalis*. The levels of genetic diversity were higher at species level (*H *=* *0.2031) but lower at population level (*H *=* *0.1096). The higher genetic diversity at species levels might be attributed to the accumulation of distinctive genotypes which adapted to the different habitats after Quaternary glaciations. Meanwhile, founder effects on the early stage, and subsequent bottleneck of population regeneration due to its biological characteristics, environmental features, and human activities, seemed to explain the low population levels of genetic diversity. The hierarchical AMOVA revealed high levels (42.60%) of among‐population genetic differentiation, which was in congruence with the high levels of Nei's genetic differentiation index (*G*_ST_ = 0.4629) and limited gene flow (*N*_m_ = 0.5801) among the studied populations. Mantel test showed a significant isolation‐by‐distance, indicating that geographic isolation has a significant effect on genetic structure in this species. Unweighted pair‐group method with arithmetic average clustering, PCoA, and Bayesian analyses uniformly recovered groups that matched the geographical distribution of this species. In particular, our results suggest that Yangtze River has served as a natural barrier to gene flow between populations occurred on both riversides. Concerning the management of *P. subaequalis*, the high genetic differentiation among populations indicates that preserving all five natural populations in situ and collecting enough individuals from these populations for ex situ conservation are necessary.

## Introduction

*Parrotia subaequalis* (Li et al. 1997, 1999; Hao and Wei 1998) has fascinated botanists for its unique systematic position within Hamamelidaceae and for its Critically Endangered status (sensu IUCN). In 1960, this species was first described as *Hamamelis subaequalis* (Hamamelidaceae) based on a single fruiting specimen collected from the Longchi Mountain, Jiangsu Province of China (Chang 1960). The species remained in obscurity until 1992 when flowering plants were discovered and a new morphological study revealed that they have a set of unique floral traits not found in other members of the Hamamelidaceae. Among these unusual morphological characters, the most distinctive one is the presence of apetalous bisexual flowers. A new monotypic genus, *Shaniodendron* (tribe Fothergilleae), was proposed to accommodate this taxon (Deng et al. 1992a). However, subsequent molecular data suggested that *Shaniodendron* is sister to the West Asian species *Parrotia persica* (Li et al. 1997, 1999). This phylogenetic placement resulted in the transfer of *S*. *subaequale* to *Parrotia* as *P. subaequalis* (Hao et al. 1996; Li et al. 1997, 1999; Hao and Wei 1998). Although *Shaniodendron* is not any longer accepted by Hamamelidaceae specialists, *P. subaequalis* is still a Critically Endangered species (Grade I Key protected Wild Plant) in the China Red Data Book, with a very narrow distribution range. The five known relict populations of *P. subaequalis* comprise no more than 100 reproductive individuals. Therefore, this is a species with a high conservation priority. To date, previous studies about *P. subaequalis* mainly focused on its phylogenetic placement, taxonomic status, morphology, distribution ranges, habitat ranges, and ecophysiological traits (Deng et al. 1992a, 1997; Li et al. 1999; Yan et al. 2008; Yao et al. 2010). However, population genetic surveys have never been reported for this species.

**Figure 1:**
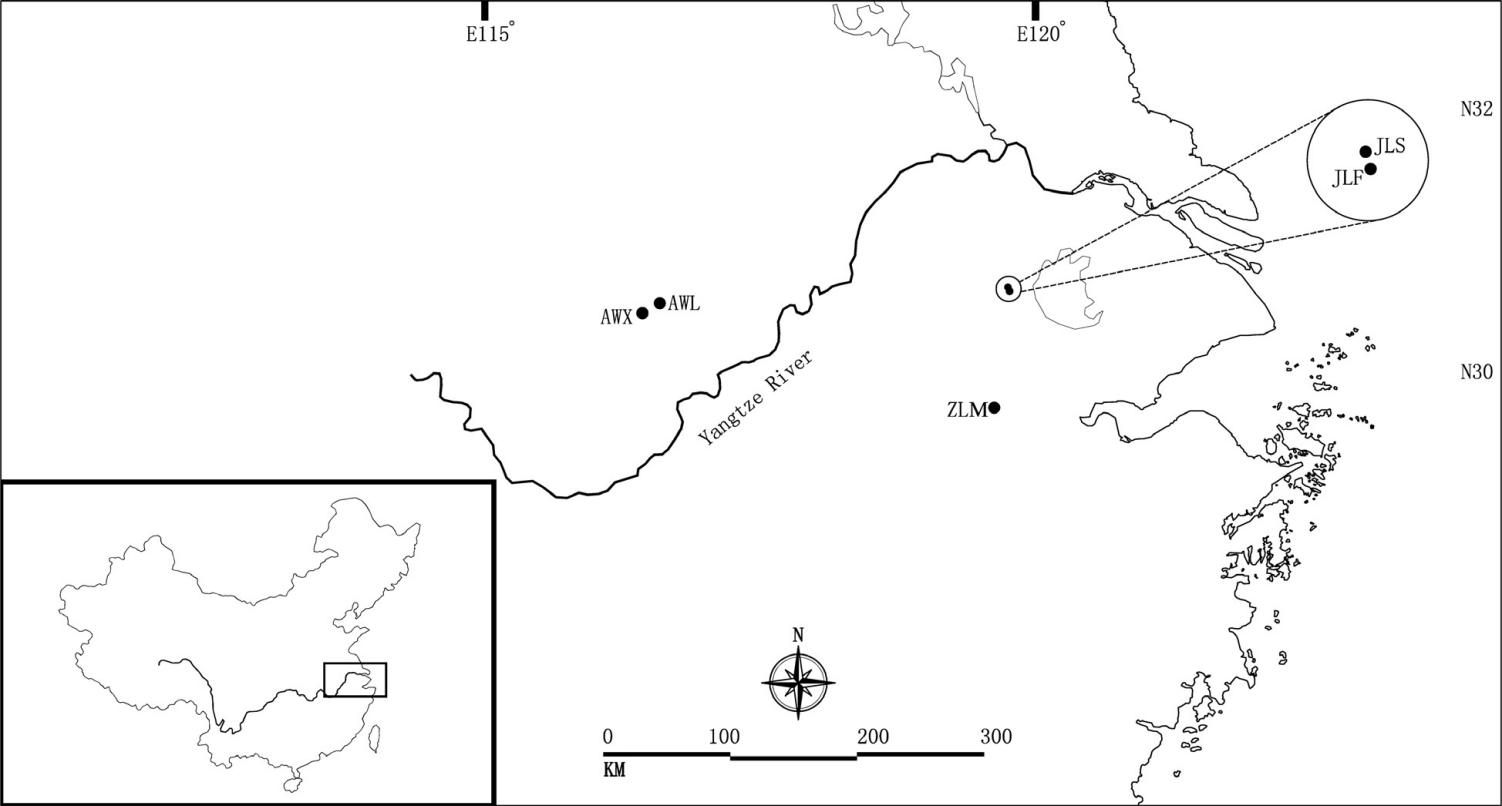
Geographic distribution of five populations of *Parrotia subaequalis* in China.

As a Tertiary relic plant, *P. subaequalis* is endemic to eastern China where it has a disjunct distribution in the Anhui, Jiangsu, and Zhejiang Provinces (Fig. 1). The five populations are distributed in the Wanfo, Longchi, and Longwang Mountains. The present mosaic distribution pattern of *P. subaequalis* has been linked to Pleistocene glaciations. The populations of *P. subaequalis* are regarded as glacial relicts as it has been suggested that these three mountains provided a glacial refugia for this species during the Quaternary Ice Ages (Hu and Chaney 1940; Deng et al. 1992b).

The Wangfo Mountain is located at the north of Yangtze River, while the Longchi and Longwang mountains are located at the south of this river. Therefore, the Yangtze River enhances geographical isolation among these populations. This river is the longest one in Asia and the third longest in the world, with a length of 6300 km running from glaciers on the Qinghai–Tibet Plateau in Qinghai across southwest, central, and eastern China before reaching the East China Sea at Shanghai.

In our study, inter‐simple sequence repeat (ISSR) markers were employed to assess the genetic diversity and population genetic structure in natural populations of *P. subaequalis*. In addition, we tested the hypothesis that Yangtze River poses additional barrier to gene flow among populations separated by this river. Finally, we offered suggestions for effective conservation and management of *P. subaequalis* based on our study.

## Material and Methods

### Study area and sampling

A total of 139 individuals from the five known populations of *P. subaequalis* were collected throughout its entire distribution region. Two populations (AWL and AWX) are distributed in Wanfo Mountain of Anhui Province, two additional populations (JLS and JLF) occur in Longchi Mountain of Jiangsu Province, and the fifth population (ZLM) is found in Longwang Mountain of Zhejiang Province (Table 1; Fig. 1). All but one of the five populations have a very small size, with less than 45 individuals each. Population JLF is an exception, with about 1400 individuals (but less than 50 reproductive plants) occupying an area of around 40 ha (Yan et al. 2008). For this population, leaves were randomly sampled from 26 trees, at least 20 m apart from one another. For the remaining four populations, leaves of all individuals were collected. Fresh leaves were collected and kept at 4°C in sealed bags then stored at −70°C until DNA extraction.

**Table 1:** Characteristics of five populations of *Parrotia subaequalis*

Location	Population	Latitude (N)	Longitude (E)	Population size^a^	Sample sizes^c^
Liujiawan, Wanfo Mountain, Anhui Province	AWL	31°07′32″	116°41′51″	38 (11)^b^	38
Xiaojianchong, Wanfo Mountain, Anhui Province	AWX	31°04′46″	116°33′54″	16 (0)	16
Shanjuandong, Longchi Mountain, Jiangsu Province	JLS	31°13′14″	119°41′54″	43 (16)	43
Forestry farm, Longchi Mountain, Jiangsu Province	JLF	31°12′31″	119°42′06″	1400 (41)	26^c^
Mafengan, Longwang Mountain, Zhejiang Province	ZLM	30°22′06″	119°32′32″	16 (8)	16

a All individuals found were counted in each population.

b Reproductive individuals in each population were counted.

c 26 individuals were randomly sampled in JLF population, including reproductive and immatured individuals.

### DNA extraction and ISSR amplification

Genomic DNA was extracted according to the modified CTAB method (Doyle and Doyle 1990). All samples that yielded clear and discernible bands after PCR amplification and electrophoresis were selected for the study. PCRs were performed in a GeneAmp 9700 DNA thermal Cycler (Perkin‐Elmer, California, USA), with a 25 *μ*L reaction mixture consisting of 1× thermostable PCR buffer (Tris‐HCl [20 mmol/L pH 8.55], (NH_4_)_2_SO_4_ [50 mmol/L], EDTA [0.1 mmol/L], Thesit [50%]), glycerol, MgCl_2_ [2.5 mmol/L]), 0.2 mmol/L each of dATP, dCTP, dGTP and dTTP, 1.5 unit of Taq DNA polymerase (Promega, Madison, WI), 0.5 mmol/L random 10‐base primer, and 40 ng genomic DNA. The PCR amplifications were run for 45 cycles at 94°C for 1 min (denaturation step), 50°C for 45 sec (annealing step), and 72°C for 5 min (elongation step), preceded by an initial melting step of 5 min at 94°C, and followed by a final extension step of 5 min at 72°C.

One hundred ISSR primers (UBC primer set No. 9, Biotechnology Laboratory, University of British Columbia) were screened for amplification, ten of which (UBC803, UBC804, UBC812, UBC823, UBC824, UBC827, UBC835, UBC836, UBC846, and UBC857) were selected for PCR amplification because they exhibited clear bands, were polymorphic, and showed reproducibility. As negative controls, we performed PCRs containing all the PCR components except DNA template.

Amplification products were separated by means of electrophoresis on 1.5 percent (w/v) agarose gels in 1× TAE buffer (40 mmol/L Tris acetate, 1 mmol/L EDTA Ph 8.0). Sizes of the amplification products were estimated by comparisons with a standard molecular weight marker (GeneRuler100 bp ladder, MBI, Vilnius, Lith). Amplified fragments were scored as presence (1) or absence (0) of homologous bands, and were then transformed into a binary matrix. Bands with a frequency of < 3/*N* (where *N* is the sample size, 139 individuals) were removed from data analyses to avoid bias in parameter estimations (Lynch and Milligan 1994).

### Data analysis

ISSR bands were scored as present (1) or absent (0) for each locus. This resulted in a data matrix that was the basis for subsequent analyses. We used the program POPGENE version 1.32 (Fc et al. 1997) to obtain the following genetic diversity parameters: the percentage of polymorphic band (PPB), Nei's unbiased genetic diversity (*H*) (Nei 1973), Shannon's index (*I*), Nei's unbiased genetic distance (*N*_*D*_) (Nei 1978), Nei's unbiased genetic identity (*N*_*I*_) (Nei 1978), total gene diversity (*H*_T_), gene diversity within populations (*H*_S_), Nei's genetic differentiation index among populations (*G*_ST_) (Nei 1973), and gene flow (*N*m). An estimate of *N*m (number of migrants per generation) value among populations was computed using the formula of *N*m = 0.5(1 − *G*_ST_)/*G*_ST_ (McDermott and Mcdonald 1993).

The significance of correlation between Nei's unbiased genetic distances and geographic distances (in km) was performed by Mantel test (Mantel 1967) with 9999 random permutations using GENALEX 6.5 software (Peakall and Smouse 2006, 2012).

The obtained genetic distance matrix was then used to perform a cluster analysis [based on the unweighted pair‐group method with arithmetic average (UPGMA)] using MEGA 5.0 (Tamura et al. 2011). Principal coordinates analysis (PCoA) was computed using GENALEX 6.5 (Peakall and Smouse 2006, 2012).

Analysis of molecular variance (AMOVA) was performed to partition the total genetic variance among populations and among geographic regions (either south of the Yangtze River or north of Yangtze River), using the AMOVA program 1.55 (Excoffier et al. 1992; Stewart and Excoffier 1996). The input files for AMOVA were prepared with AMOVA‐PREP 1.01 (Miller 1998). Significance tests were made after 1000 permutations.

Bayesian methods were also utilized to recover the optimal number of clusters that compose the studied populations. These Bayesian analyses were implemented using STRUCTURE 2.3 software (Pritchard et al. 2000; Falush et al. 2007). Twenty independent runs for each K (from 1 to 5 clusters) were performed using 1 000 000 MCMC (Markov Chain Monte Carlo) repetitions and 100 000 burn‐in periods (Gilbert et al. 2012), using no prior information and assuming correlated allele frequencies and admixture in this study. Results obtained from STRUCTURE were further interpreted by STRUCTURE HARVESTER (Earl and Vonholdt 2012), which implements Evanno's Δ*K* method (Evanno et al. 2005) for calculation of the optimal *K*. To obtain optimal alignment of the independent runs, the CLUMPP version 1.1 (Jakobsson and Rosenberg 2007) were used with greedy algorithms, 10,000 random input orders and 10,000 repeats, to calculate the average pairwise similarity (*H*′) of runs. Finally, the clustered output was visualized using the software Distruct version 1.1 (Rosenberg 2004).

## Results

### Genetic diversity of *Parrotia subaequalis*

Across all 139 *P. subaequalis* individuals form the five populations, a total of 108 reproducible bands were presented from the ten ISSR primer set. Of the 108 bands surveyed, 74 (68.52%) of these loci were polymorphic among the sampled populations. At the population level, the mean values of the Nei's gene diversity, Shannon information index, and the percentage of polymorphic loci were 0.1096, 0.1698, and 36.11%, respectively. The JLF population (*H *=* *0.1376, *I *=* *0.2120, PPB* *= 44.44%) in Longchi Mountain had the highest genetic diversity, while the ZLM population (*H *=* *0.0841, *I *=* *0.1310, PPB* *= 26.85%) in Longwang Mountain had the lowest genetic diversity. At the species level, the Nei's gene diversity, Shannon information index, and the percentage of polymorphic loci were 0.2031, 0.3132, and 68.52%, respectively (Table 2).

**Table 2:** Genetic diversity within the populations of *Parrotia subaequalis* detected by ISSR analysis

Population	*N*	*N*_*a*_	*N*_*e*_	*H*	*I*	PPB (%)
AWL	38	1.3796	1.1738	0.1097	0.1710	37.96
AWX	16	1.2685	1.1382	0.0857	0.1315	26.85
JLS	43	1.4444	1.2087	0.1311	0.2033	44.44
JLF	26	1.4444	1.2199	0.1376	0.2120	44.44
ZLM	16	1.2685	1.1295	0.0841	0.1310	26.85
Mean		1.3611	1.1740	0.1096	0.1698	36.11
Species level	139	1.6852	1.3335	0.2031	0.3132	68.52

*N*, number of samples; *N*_*a*_, observed number of alleles; *N*_*e*_, effective number of alleles; *H*, Nei's (1973) gene diversity; *I*, Shannon's information index; PPB, percentage of polymorphic bands.

### Genetic differentiation among populations and gene flow

The total gene diversity (*H*_T_ = 0.2042 ± 0.0342) was primarily distributed among populations (*H*_S_ = 0.1096 ± 0.0110). The value of Nei's genetic differentiation index among populations (*G*_ST_) was 0.4629, indicated that a relatively high level of genetic differentiation existed among the five populations. The hierarchical AMOVA revealed that 42.60% of the total variation was attributed to differences among five populations and that 57.40% was contributed by differences within populations (*P *<* *0.001) (Table 3). Based on the *G*_ST_ value, the estimated number of migrants per generation (*N*_m_) was 0.5801.

**Table 3:** Analysis of molecular variance (AMOVA) within/among *Parrotia subaequalis* populations and within/among geographic regions^a^

Source of variation	df	SSD	MSD	Variance component	Total variance	*P*‐value
Among Populations	4	683.56	170.89	6.09	42.60%	<0.001
Within Populations	134	1102.04	8.22	8.22	57.40%	<0.001
Among geographic regions	1	288.82	288.82	4.2	27.80%	<0.001
Within geographic regions	137	1496.78	10.93	10.93	72.20%	<0.001

a Geographic regions are south of Yangtze River (JLS, JLF and ZLM) and north of Yangtze River (AWL, AWX).

df, degrees of freedom; SSD, sum of squared deviation; MSD, mean squared deviation; *P*‐value, probability. Significance tests after 1000 random permutations.

For the AMOVAs, populations of *P. subaequalis* were also assigned to two geographic regions: south of Yangtze River (JLS, JLF, and ZLM), and north of Yangtze River (AWL, AWX). The AMOVA revealed that 27.80% of the total variation was distributed among geographic regions, whereas 72.20% was found within geographic regions (*P *<* *0.001) (Table 3).

### Genetic relationships

Populations sampled from the same mountain were more similar to each other than those from different mountains. Genetic analysis showed that the highest identity value (*N*_*I*_ = 0.9154) existed between populations JLS and JLF in the Longchi Mountain (Table 4). In contrast, the AWX population in the Wanfo Mountain, which occurs at the north of Yangtze River, and the ZLM population in the Longwang Mountain, which is located at the south of Yangtze River, showed the lowest genetic identity values (*N*_*I*_ **= 0.8031) (Table 4). Accordingly, the highest genetic distance value was 0.2193 (between populations AWX and ZLM), while the lowest genetic distance value (0.0884) was between populations JLS and JLF (Table 4).

**Table 4:** Nei's (1978) unbiased measures of genetic identity (above diagonal) and genetic distance (below diagonal) among populations of *Parrotia subaequalis* based on ISSR analysis

Pop ID	AWL	AWX	JLS	JLF	ZLM
AWL	‐	0.8959	0.8486	0.9087	0.8406
AWX	0.1100	‐	0.8258	0.8869	0.8031
JLS	0.1642	0.1915	‐	0.9154	0.8852
JLF	0.0958	0.1200	0.0884	‐	0.8902
ZLM	0.1737	0.2193	0.1219	0.1163	‐

**Figure 2:**
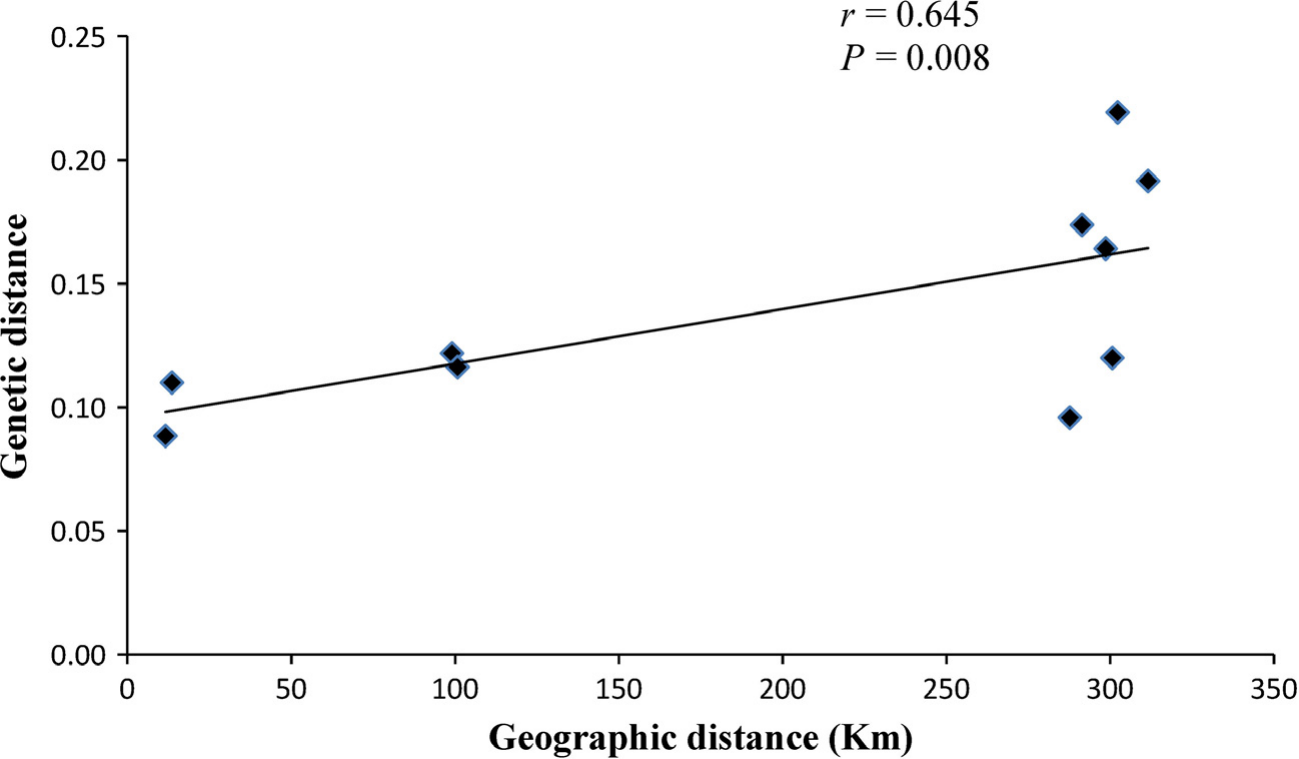
The correlation between Nei's unbiased genetic distance and geographical distance for five populations of *Parrotia subaequalis*.

The Mantel's test results further indicated that there was a significant correlation (*r *=* *0.642, *P *=* *0.008; *P *<* *0.001, Fig. 2) between Nei's unbiased genetic distance and geographical distance among the five populations. This suggested that the differentiation observed among populations directly corresponded to the geographic distance.

**Figure 3:**
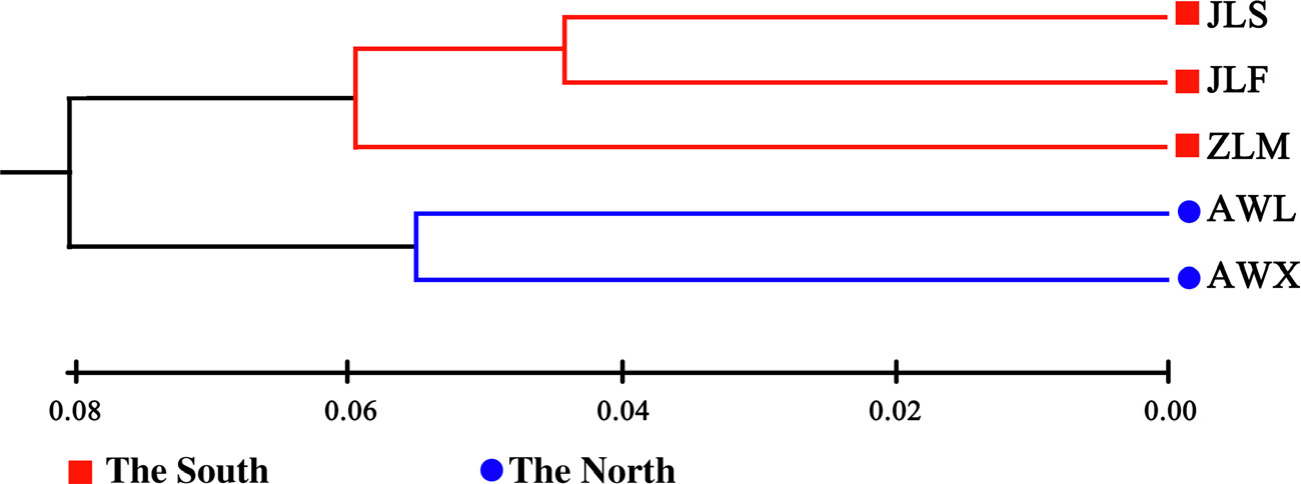
Unweighted pair‐group method with arithmetic average dendrogram based on Nei's (1978) unbiased genetic distances among the populations of *Parrotia subaequalis*.

The UPGMA dendrogram based on Nei's unbiased genetic distance clustered all populations into two major groups (Fig. 3). As expected, these two groups were in accordance with the major geographical areas where the species is distributed. One group has three populations located at the southern of Yangtze River, whereas the second group has two populations found at the northern of the Yangtze River. Similarly, populations sampled from the same mountain, such as AWL and AWX (both in Wanfo Mountain), JLS and JLF (both in Longchi Mountain) clustered into two different groups that match their geographical provenance.

**Figure 4:**
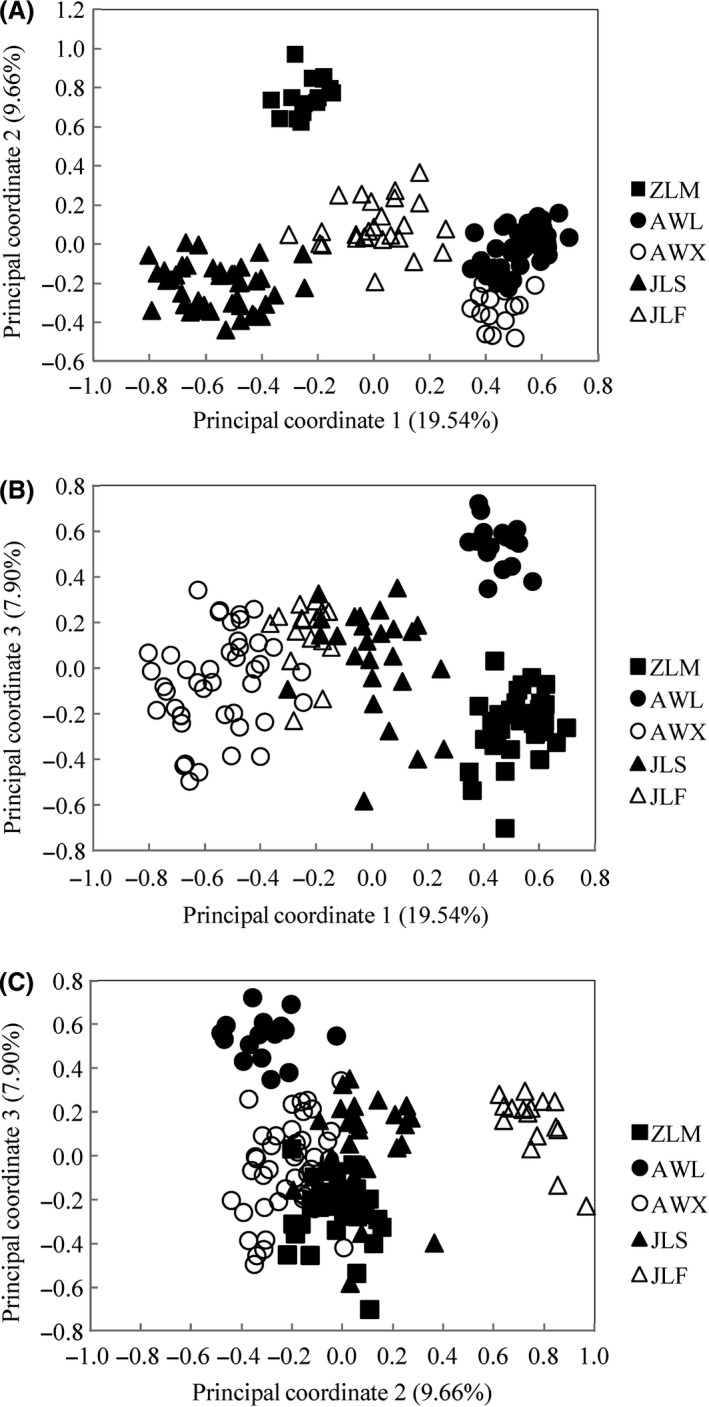
Two‐dimensional plot of the principal coordinates analysis (PCoA) from the ISSR data of 139 individuals for *Parrotia subaequalis*. (A) Plot of principal coordinates 1 vs. 2; (B) Plot of principal coordinates 1 vs. 3; (C) Plot of principal coordinates 2 vs. 3.

The Principal coordinate analysis (PCoA) generated three axes with interpretable patterns (Fig. 4). The first three components of PCoA accounted for 19.54% (Axis 1), 9.66% (Axis 2), and 7.90% (Axis 3) of total variance among populations, respectively (Fig. 4). The five populations formed their own groups. The populations in the same Mountain had similar values in the PCoA scatter diagram. The principal component analysis was in agreement with the UPGMA dendrogram and clustered all of the individuals into three groups based on their geographical origin.

**Figure 5:**
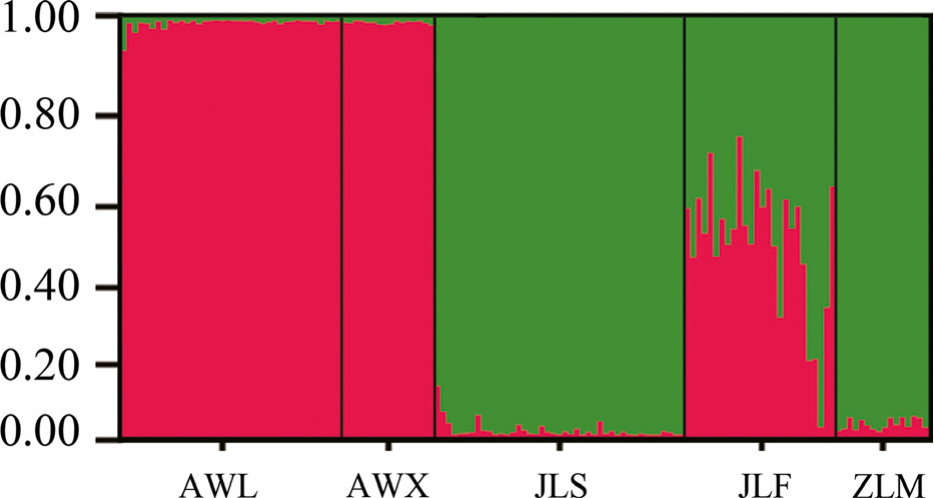
Genetic relationships among the five populations of *Parrotia subaequalis* estimated using STRUCTURE program based on ISSR data.

In the ISSR admixture analysis using STRUCTURE, the real *k* value with the highest value of Δ*K* for the 139 individuals was *K *=* *2 (Fig. 5). The proportions of each individual in each population were assigned into two clusters (cluster I and cluster II) (Fig. 5). This result is in agreement with UPGMA dendrogram. However, JLF population displayed some degree of mixed ancestry.

## Discussion

### Genetic diversity of *Parrotia subaequalis*

There is evidence that plant species with narrow distributions generally have low levels of genetic diversity (Hamrick and Godt 1989). *Parrotia subaequalis* is an endemic and relict species restricted to three Mountains in eastern China. However, our ISSR survey of its five natural populations revealed a relatively high level of genetic diversity at the species level (PPB = 68.52%, *H *=* *0.2031, *I *=* *0.3132; Table 2). Interestingly, other species in the same family also have high genetic diversity at the species level, such as *Distylium chinense* (*H*_ISSR_ = 0.2379, *H*_SRAP_ = 0.2159) (Li et al. 2011; Xie et al. 2012), *Liquidambar fornosana* (*H*_pop_ = 0.3122) (Bi et al. 2010), and *Mytilaria laosensis* (*H*_ISSR_ = 0.2969) (Peng et al. 2012). The Hamamelidaceae belong to an early branch of the Angiosperms (Hu et al. 2011), and the high genetic diversity detected in these studies might be in part the result of this phylogenetic placement as an ancient family (Bi et al. 2010; Peng et al. 2012).

When *P. subaequalis* is compared with other endangered and endemic species with same life‐history traits, the genetic diversity at the species level is also higher (Hamrick and Godt 1996). As compared with *Astragalus nitidiflorus* (Leguminosae; PPB = 51.3%, *H *=* *0.1712, *I *=* *0.2608, at species level) (Vicente et al. 2011), *Emmenopterys henryi* (Rubiaceae; PPB = 56.05%, *H *=* *0.191, *I *=* *0.287, at species level) (Li and Jin 2008), and *Dysosma versipellis* (Berberidaceae; PPB = 57.64%, *H *=* *0.21, *I *=* *0.31, at species level) (Qiu et al. 2006), *P. subaequalis* also exhibits relatively high levels of genetic diversity at the species level. Thus, *P. subaequalis* belongs to a minority of endemics maintaining high levels of genetic diversity (Smith and Pham 1996).

The genetic diversity of *P. subaequalis* was high at the species level and a large proportion of variation resided among populations. ISSR markers have yielded similar results with some other endemic plants [i.e., *Sinojackia dolichocarpa* (Cao et al. 2006), *Gynostemma pentaphyllum* (Wang et al. 2008), and *Magnolia officinalis* (Yu et al. 2011)], suggesting that particular historical and biological factors can in some cases influence a particular threatened species to avoid genetic erosion through stochastic events. Factors such as Pleistocene refugia effect, somatic mutations, or multiple founder events have been invoked to explain unexpected levels of high genetic diversity at species level in rare plants (Richter et al. 1994; Smith and Pham 1996; Ge et al. 1997; Zawko et al. 2001; Gonzalez‐Astorga and Castillo‐Campos 2004; Cao et al. 2006; Yu et al. 2011). The Wangfo, Longchi, and Longwang mountains are considered as biodiversity refugia that have been harboring residual populations since the Tertiary (Hu and Chaney 1940; Deng et al. 1992b). The higher genetic diversity at species level may result from the accumulation of different genotypes which adapted to the different habitats in three mountains between two sides of the Yangtze River.

By contrast, the genetic diversity of *P. subaequalis* was relatively low at population level. It is well known that historical events may contribute to patterns of genetic diversity of organisms (Karron 1991). *Parrotia subaequalis* might be an example of those Asian plants suggested to follow a migration path from northern to southern latitudes during the Quaternary glaciations [see (Chen et al. 2011)]. One of the suggested migration corridors runs from northern areas to Tianmu across the Dabie Mountain System (Deng et al. 1992b). During this migration process, each population of *P. subaequalis* may have experienced severe founder effects, bottlenecks and subsequent genetic drift and inbreeding that led to a decline of genetic diversity at population level.

It is likely that the genetic consequences of these historical events have been enhanced by the biological characteristics of *P. subaequalis*. This species blooms every two to three years (Hu et al. 2011), and often encounters cold snaps and rain showers during its flowering season (Deng et al. 1997). In addition, this species has a very low fruit setting rate, a high rate of underdeveloped seed (20.3%), and a low germination rate (17–19%) (Deng et al. 1997; Zhang et al. 2011). Therefore, these processes might limit population regeneration and expansion, which eventually may also have a negative influence on the maintenance of genetic diversity at the population level.

Based on our field observations, it appears that several environmental features are major obstacles for the long‐term preservation of *P. subaequalis* and might also have a detrimental effect on the effective population size of this species. The known populations are located along mountain streams, slopes, and rocky soils that appear to be nutrient‐poor; therefore, they do not provide the best environment for seed germination and seedling establishment (Gong et al. 2012; Ren et al. 2012). Furthermore, these populations are under severe competition with other species, especially with bamboos (JLF population in Longchi Mountain). These other species not only deprive *P. subaequalis* of needed resources but result in an environment in which the understory is under reduced light conditions. It has been found that *P. subaequalis* does not grow well under shade (Liu and Hao 1999; Liu et al. 2008b), and therefore, it is believed that this is one of the main factors that account for its threatened status (Fang et al. 2004).

Human activities in the areas where our target species grows also appears to contribute to the reduced levels of within‐population genetic diversity. The three mountains where *P. subaequalis* is found are important tourist destinations, and this has resulted in disturbed habitats that are not suitable for the expansion of this species. Furthermore, clearance of forest understory is part of the land management procedures in this region, and this practice has resulted in reduced recruitment as many seedlings are removed. Due to these harmful human interventions, populations of AWX and ZLM with smaller size had relatively lower genetic diversity.

As a summary, historical events, biological characteristics, environmental features, and human activities have contributed to the low levels of genetic diversity at the population level.

### Population genetic structure

In our study, both Nei's genetic differentiation index among population (*G*_ST_ = 0.4629), and AMOVA (42.6%) values indicated significant genetic differentiation among the studied populations. The *G*_st_ values observed for *P. subaequalis* are above the averages observed for endangered and endemic plants with same life‐history traits (Hamrick and Godt 1996; Nybom 2004). Similar results have been reported in other endangered or endemic species, such as *Rheum tanguticum* (Polygonaceae, *G*_ST_ = 0.497) (Wang et al. 2012) and *Megacodon stylophorus* (Gentianaceae, *G*_ST_ = 0.727) (Ge et al. 2005). The high genetic differentiation exhibited by *P. subaequalis* can be explained by a disjunct distribution because of geographic isolation that has resulted in limited gene flow (Schaal et al. 1998; Hao et al. 2006).

Natural barriers such as rivers, shorelines, mountains, or glaciers can restrict or prevent gene flow and result in genetic differentiation among populations (Lecorre et al. 1997; Nesbo et al. 1998; Su et al. 2003). As a natural barrier, the Yangtze River is considered to have played a major role in the evolution and biogeography of the Chinese flora (Wu 1983). Phytogeographical data indicate that it has been an important geographical barrier to plant migration between its two riversides (Huang et al. 2003; Zhang et al. 2007). In our study, AMOVAs revealed that 27.80% of the total variation was distributed among geographic regions, which could be explained by the natural geographic area effect of Yangtze River. Based on molecular markers, similar results were found in other plants with same life history such as *Vitex negundo* (Verbenaceae) and *Schisandra sphenanthera* (Magnoliaceae) (Zhang et al. 2007; Yan et al. 2009).

Bayesian analysis for the optimal *K *=* *2 (Fig. 5) also showed that all sampled individuals of *P. subaequalis* were assigned to two different clusters. Individuals from sites located north of the Yangtze River belonged primarily to one of these clusters. Those from populations situated south of this river were predominantly assigned to the second cluster. It should note that JLF population display some degree of mixed ancestry, which may result from the migration from northern to southern latitudes during the Quaternary glaciations (Chen et al. 2011). In addition, founders of this population most probably had a different genetic constitution. Unweighted pair‐group method with arithmetic average cluster analysis and PCoA yielded genetic clusters also revealed this genetic differentiation caused by the Yangtze River.

Mantel test showed a significant isolation‐by‐distance (IBD) pattern in *P. subaequalis*, indicating that geographic isolation has a significant effect on the genetic structure in this species. The smallest distances between the mountains where this species occur is 99 km, where as the largest is 312 km, which largely impeded the exchange of seeds or pollen among sites in different mountains. Seeds of *P. subeaqualis* are small, and thousand seed weight, single seed length and width have been reported to be about 32.7 g, 7 mm and 3.5 mm, respectively (Liu et al. 2008a; Hu et al. 2011). These light‐weight seeds can be ejected and spread with the elasticity of the capsule, for as far as 18 m (Yang 1994; Deng et al. 1997). However, there is no obvious trait to suggest seed dispersal by vertebrates or another other long‐distance seed dispersal mechanisms. Lack of effective mechanisms for long‐distance dispersal of seeds may play an important role in the observed high levels of genetic diversity among populations. Although pollen of *P. subaequalis* is dispersed by wind, the mountains where this species occur are distant from each other. Therefore, it does not seem plausible that gene flow through pollen migration is a regular ecological process of these populations.

### Conservation implications

An understanding of the genetic structure and diversity of populations is important for establishing conservation strategies for relict and endangered species (Hamrick and Godt 1989). The conservation of *P. subaequalis* genetic resources involves not only preventing extinction but also ensuring the availability of resources for the changing environments in the future. Currently, all known populations are protected (Wang et al. 2001; Fang et al. 2004; Zhang 2014). However, more proactive conservation actions may be needed. For example, two of the five populations have less than 20 individuals and fewer are of reproductive size. Small populations like these may be prone to local extinction and may benefit from population augmentation. Considering the significant differences between populations, we suggest the use of source material from the same site or nearby site when carrying out augmentation of these populations. Another measure that should be taken is enhancing habitat quality of *P. subaequalis* via active habitat management. For example, population of *P. subaequalis* in Longchi Mountain appeared to be negatively impacted by overcrowded bamboos and other associate tree species (Yan et al. 2008). Thinning bamboos and other associate tree species may be needed to prevent population decline at this site.

In addition to efforts to preserve all the extant populations and their habitats, we recommend establishment of comprehensive ex situ collections in botanical gardens, which represent the genetic diversity in the wild. In order to maintain the suitability of these ex situ materials as sources for population augmentation and reintroduction (in the event of local extinctions), it is desirable to keep ex situ collections represent the north and south populations of the Yangtze River and far apart populations separate, to prevent unwanted crosses among these populations in the botanical gardens (Zhang et al. 2010). Furthermore, artificial pollination is necessary to generate seeds needed from the wild populations for ex situ collections. This is due to the fact that natural seed set and seed germination rates were low (Deng et al. 1997; Li et al. 2012). Supplemental artificial pollination may also be a tactic to augment in situ populations.

Our study represents only part of a comprehensive conservation research plan that may lead to the long‐term survival of the species. Other research needed includes a long‐term population monitoring and dynamic study as well as the species's habitat requirement, which will lead to better management measures that guarantee the long‐term viability of the species.

## Conflict of Interest

None declared.
